# Performance Progression and Stability of Female Swimmers Across Different Swimming Techniques from Childhood to Adulthood

**DOI:** 10.3390/sports14040164

**Published:** 2026-04-21

**Authors:** Francisco A. Ferreira, Mário J. Costa, Catarina C. Santos

**Affiliations:** 1 Centre of Research, Education, Innovation and Intervention in Sport, CIFI2D, Faculty of Sport, University of Porto, 4200-450 Porto, Portugal; 2 Porto Biomechanics Laboratory, LABIOMEP, Faculty of Sport, University of Porto, 4200-450 Porto, Portugal; 3 Department of Sport Sciences, Exercise and Health, University of Trás-os-Montes and Alto Douro, 5000-801 Vila Real, Portugal; mariocosta@utad.pt (M.J.C.); catarinasantos@utad.pt (C.C.S.); 4 Research Center in Sports Sciences, Health and Human Development, CIDESD, 5000-801 Vila Real, Portugal; 5 Higher Education School, Polytechnic of Coimbra, 3030-329 Coimbra, Portugal; 6 SPRINT Sport Physical Activity and Health Research & Innovation Center, 3030-329 Coimbra, Portugal

**Keywords:** swimming, performance, tracking, long-term athlete development, talent identification

## Abstract

The aim of this study was to understand the female swimmers’ annual performance progression and stability between 10 and 18 years across swimming distances and techniques. Data from female Portuguese Top-50 rankings in the short-course pool was extracted from an open access database (swimrankings.net). Performances were grouped by distances (50-, 100- and 200 m) and techniques (freestyle, backstroke, breaststroke and butterfly), totalizing 12 events as performance metrics. A total of 343 swimmers and 3087 performances distributed by nine consecutive competitive seasons were retrospectively assessed. The mean and normative stability were computed for tracking performance trends, while reporting the year-to-year percentage improvement. The differences across distances and techniques were tested with a linear mixed-effects model using intraclass correlation coefficient (ICC). The performance progression was characterized by marked improvements during the early ages (up to 13% yearly) and an emerging plateau around the 15–16 years. The stability patterns varied between events, with the backstroke technique (ICC = 0.13) demonstrating greater consistency of individual differences on developmental trajectories, whereas shorter races (i.e., 50 m; ICC = 0.15) tended to be more stable than 100 m or 200 m (ICC = 0.12). It can be concluded that female swimmers’ performance stabilizes at the 15–16 years of age. Despite reduced differences, the backstroke technique and short distances seem to show a slightly more stable trend in progressing from childhood to adulthood.

## 1. Introduction

Tracking annual performance progression from childhood to adulthood is crucial to understanding how athletes progress throughout their career [1] and how they may improve in the long-term [2]. Achieving sport success in adulthood may be influenced by both nature (e.g., sex, anthropometric and maturation [3,4,5]) and nurture factors (e.g., training characteristics and technical skills acquisition [6]). This pathway contributes to some (non)fluctuations in performance. In the specific case of swimming, the performance progression varies between sex, swimming strokes and race distances [7], with stability patterns yet to be clearly defined.

Stability of swimming performance (i.e., the consistency of an athlete’s ranking or achievement over time) plays a crucial role in early identification of talent as it allows coaches, athletes and researchers to predict future performance [8]. Swimmers reach stability as they approach their peak performance [9] and this may determine their presence in national squads [10]. Evidence suggests that male swimmers typically stabilize their performance around 16 years of age [1], but this does not mean that, in some cases, performance may still decline or improve in the following years. For instance, European elite swimmers show a continuous time decline in annual progression, with females plateauing earlier than males (12–13 vs. 14–15 years, respectively [11]). Despite apparent sex-related differences in the timing of stabilization, childhood performance does not seem to be a good predictor for adult performance, at least for European sprint swimmers [3]. However, available research has predominantly focused on male swimmers [1,12] and/or freestyle events [1,11,12,13,14].

Identifying the factors that contribute to the progression of female performance during the transition from childhood to adulthood remains complex, especially given the variations in the puberty onset [15]. Several models have been presented for this multifactorial phenomenon. However, the determinants for female swimmers in 400 m freestyle seem to be highly dependent of foot length and body fat percentage [16]. Thus, understanding the progress throughout their careers across all four competitive techniques, and in the various race distances, may allow for deeper insights and define benchmarks [15,17] of average annual performance progression and stabilization of performance.

Although several procedures for tracking and assessing stability have been applied in the past [8,18,19,20], two of the most common approaches are mean stability and normative stability [2,21,22]. While mean stability examines whether individuals maintain their relative position in a distribution over time, normative stability investigates the consistency of inter-individual differences in intra-individual changes. The longitudinal tracking analyses based on transition matrices (i.e., yearly time improvements and percentages) and correlation coefficients are also often used to quantify the degree to which early performance associates with later outcomes [12,23]. Moreover, but yet little explored, the linear mixed-model effects were used as a tool to make comparative analyses across swimming distances [11]. Using this approach, it is possible to provide an indirect comparison of techniques. While these statistical analyses provide valuable insights, retrospective longitudinal research simultaneously considering the interaction between swimming techniques and distances across the maturational development remains scarce. This gap in the literature highlights the need to better characterize the trajectories of female swimmers across different competitive events.

The aim of the current study was three-fold: (i) to understand the annual performance progression and analyze stability performance of female swimmers from childhood to adulthood (10 to 18 years); (ii) to compare the stability on different swimming techniques (freestyle, backstroke, breaststroke and butterfly); and (iii) to check for the existence of stability differences across distances of swimming events (50-, 100- and 200 m). It was hypothesized that there would be a lack of stability in the early years, emerging in later age-groups, and that event characteristics (technique and distances) would influence the degree of stability observed.

## 2. Materials and Methods

### 2.1. Participants

The Top-50 Portuguese female swimmers across each event (distance x technique) from short-course races (25 m pool length) were retrieved. The inclusion criteria for each competitive event comprised: (i) ranked up to 50th place; (ii) aged at least 18 years during the 2024–2025 season; and (iii) performed the event at least seven seasons (out of nine) from 10 to 18 years. Whenever necessary, statistical modelling was conducted to predict values between two known years of performance using non-linear interpolation. Multiple regression models (exponential and polynomial) were tested, selecting the best fit based on the lowest Mean Squared Error. Swimmers were excluded if they were not aged 18 during the 2024–2025 season. A total of 343 Portuguese female swimmers were included for further analysis.

### 2.2. Study Design

An observational retrospective design was selected for the present study. The Portuguese females’ Top-50 ranking for each event from the 2024–2025 season and the performances across the nine consecutive ages (best performance in each competitive season from ages 10 to 18) were consulted through the publicly accessible SwimRankings database (swimrankings.net [24]) and analyzed anonymously. A competitive season was considered from the 1 September of each year to the 31 August of the consequent year (e.g., 1 September 2024 to 31 August 2025). Swimmers’ characterization was also provided through the World Aquatics Points Scoring (pts; expressing performances relative to the prevailing World Record) through the best performance achieved among the included ages for each event [25]. A total of 3087 performances during the nine consecutive seasons (i.e., corresponding from 10 to 18 years) distributed across the 12 events (50-, 100- or 200 m x freestyle, backstroke, breaststroke or butterfly) were analyzed. The Institutional Ethics Committee stated that ethics approval was not required for this type of study design.

### 2.3. Statistical Analysis

The Kolmogorov–Smirnov test proved normality of data for all ages. The longitudinal analysis was conducted using mean stability and normative stability [1,26,27]. Mean stability (i.e., the consistency of group-level performance over time) was assessed using the quartiles distribution and mean ± standard deviation (SD) values for all ages and events. After all assumptions were met, the data were analyzed using repeated-measures ANOVA with a Bonferroni-adjusted post hoc test. Eta squared (η^2^) was used as a measure of effect size and considered [28]: no effect if η^2^ ≤ 0.04, minimum effect if 0.04 < η^2^ ≤ 0.25, moderate effect if 0.25 < η^2^ ≤ 0.64 and strong effect if η^2^ > 0.64. Complementarily, the percentage of change for consecutive ages and overall improvement across the nine seasons was used to characterize the annual performance progression. Normative stability (i.e., the consistency of an individual’s rank-order position relative to their peers) was evaluated through the Pearson correlation coefficient (r). The correlation was performed across the nine chronological ages, and the values were interpreted as [18]: high if r ≥ 0.60, moderate if 0.30 ≤ r < 0.60, and low if r < 0.30.

A linear mixed-effect model (LMM) with fixed intercepts and restricted maximum likelihood estimation was applied to compare the stability of performance on the different competitive events. The analysis was conducted according to: (i) a general model including all data to assess the overall effects of age and the interaction between distance and technique on performance stability, and (ii) separate models to compare stability across different distances and techniques. In all models, swimmers’ performance was used as the dependent variable, while age, distance, technique and their interaction were included as fixed factors (swimmer used as a random effect to account for repeated measures within individuals). Model assumptions, including linearity, normality of residuals, and homoscedasticity, were verified through visual inspection of residual plots and Q–Q plots, which confirmed that the data met the requirements for LMM.

All statistical analyses were performed using IBM SPSS software (v. 30.0.0.0, 2025, IBM Corp., Armonk, NY, USA) and significance was set at *p* < 0.05. Graphics were generated using Python (v. 3.13, 2025, Python Software Foundation, Beaverton, OR, USA) and relevant libraries (NumPy, pandas, matplotlib, seaborn) implemented in the Visual Studio Code software (v. 1.100.2, 2025, Microsoft Corporation, Redmond, WA, USA).

## 3. Results

**Figure 1:**
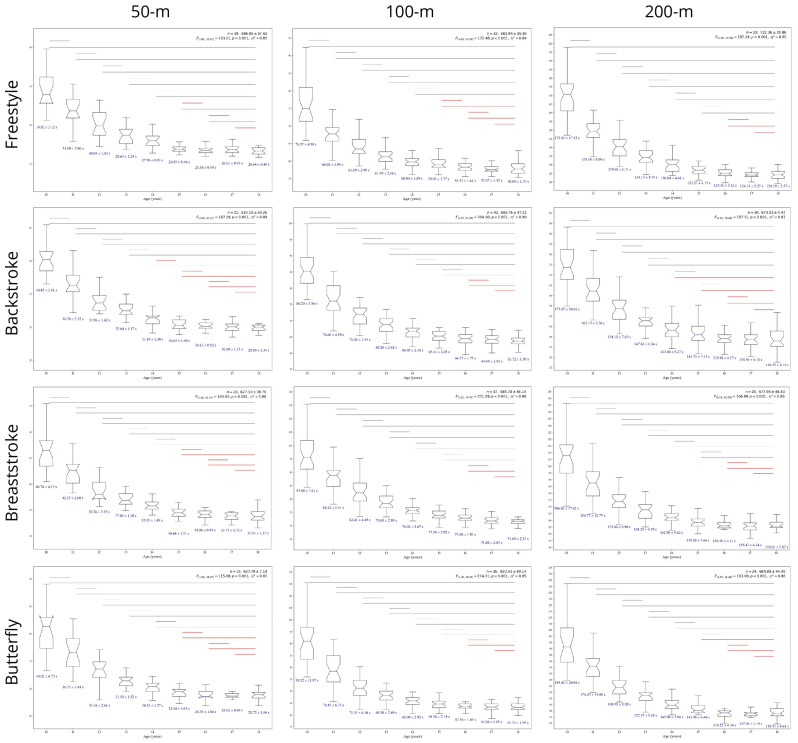
Boxplots illustrating performance distributions of female swimmers across ages 10–18 years for each competitive event. Mean ± standard deviation performance (s) values are shown in blue. Repeated-measures ANOVA is given in the top right for each event, while Bonferroni post hoc comparisons are indicated by horizontal lines. Note: F—F-test; η^2^—effect size (eta squared). Horizontal lines denote pairwise differences: dark grey (*p* < 0.001), light grey (*p* < 0.05), and red (*p* ≥ 0.05).

Figure 1 and Table S1 show variations in performances with strong effects across nine consecutive ages in all swimming events. Bonferroni post hoc highlights that the mean stability of performance depends on distance and technique, occurring at 14 (200 m backstroke), 15 (50- and 100 m freestyle, 50- and 100 m backstroke, 50- and 200 m breaststroke, and 50- and 200 m butterfly) or 16 years (200 m freestyle, 100 m breaststroke, and 100 m butterfly). Performance seems to improve yearly across all events through the time decline (s).

There were marked changes in performance during the early years (i.e., 10–15), evidenced by larger year-to-year improvements (Table 1). Contrarily, a decrease in the percentage of changes between consecutive years in the older age groups (from age 15 onwards) was found across all events. The overall improvement (i.e., 10–18 years) varied between ~22–30% with the 100 m butterfly event showing the highest change (30.89 ± 7.47%). For better visual inspection, Appendix A shows the trend towards more homogeneous performance up to age 18 through spaghetti graphs/plots.

**Figure 2:**
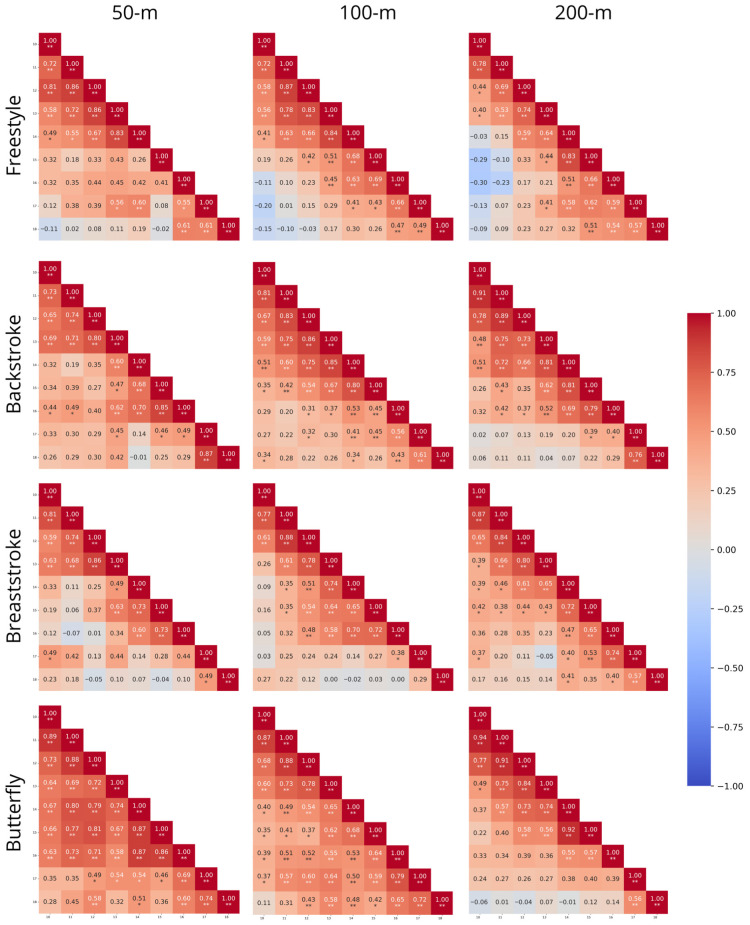
Heatmap of correlations with corresponding *p*-values across ages 10–18 years in female swimmers for each competitive event. Note: r—Pearson’s correlation coefficients; *—*p* < 0.05; **—*p* < 0.01.

Figure 2 presents the normative stability for each event, where lighter or darker colors translate into reduced or increased correlation across the different ages (respectively). Moderate to high correlations were observed between 16- and 17–18 years for 50 and 100 m freestyle, 100 m backstroke, 200 m breaststroke and 50 m butterfly. Additionally, 15 years was moderately correlated with 18 years in 200 m freestyle. 50 and 200 m backstroke, 50 m breaststroke and 200 m butterfly just correlated at 17–18 years, while 100 m butterfly correlated from 12 to 18 and on. The 50 m butterfly event lacked consistency in correlation and did not present robust stability across the age groups.

The general mixed-effects model explained 98% of the variance in performance (r-squared conditional, R^2^c = 0.98). The intraclass correlation coefficient (ICC) was 0.05, indicating that only 5% of the variance in performance was attributable to differences between swimmers after accounting for age, distance and technique. All fixed effects, namely age (F = 3029.37), distance (F = 62,993.40), technique (F = 875.00) and their interaction (F = 129.27) were significant (*p* < 0.001). Table 2 presents the separate models comparing stability across different distances and techniques, showing that stability varies according to both factors. Although the variances appeared to differ when comparing the two components, the swimming technique explained 97–99% (R^2^c = 0.99 for backstroke, R^2^c = 0.98 for breaststroke and R^2^c = 0.86 for freestyle and butterfly) of the variance in the models, whereas distance accounted for 86–87% (R^2^c = 0.87 for 50- and R^2^c = 0.86 for 100- and 200 m). Despite an overall reduction across all events, stability was highest for 50 m events (ICC = 0.15) and for backstroke events (ICC = 0.11), indicating that stable intra-individual differences in inter-individual consistency explained 15% and 11% of the variance (respectively). All effects were *p* < 0.001.

## 4. Discussion

The present study aimed to examine annual performance progression and stability profiles in female swimmers between 10 and 18 years across different techniques and distances. The annual performance progression was characterized by substantial improvements during the early years, followed by a plateau emerging around the ages of 15–16 years across the multiple events (i.e., 50-, 100- and 200 m x freestyle, backstroke, breaststroke and butterfly). While variations in technique and distance did not demonstrate marked trends or even high coefficients, performance stability tended to be slightly greater in 50 m and in backstroke events.

Previous studies on the annual performance progression from childhood to adulthood showed that swimmers tended to present performance variations across freestyle events [1]. At least for Olympic swimmers’, performance across their careers indicates a quadratic trend (initial improvement, peak performance, and subsequent time decrease [29]). Although the peak performance was not considered, the performance of female swimmers seems to follow the same tendency of improvement until the age of 18. Indeed, peak swimming performance tends to be achieved around the age of 20 years [9], with shorter-distance events often maturing later at 22–23 years [29] than middle- and long-distance races [2,7].

Also, improvements in World Records over the decades have been reported, with World Record holders becoming older, mainly in short-distance events [2]. Therefore, one may argue that the present cohort showed a similar trend (curve, not straight-line trend; see Appendix A); however, they may not have yet reached the “performance window” required to achieve their peak performance. Also, from 10 to 18 years, performance of female swimmers varied between ~8 s (freestyle) and ~12 s (breaststroke) on the 50 m, ~18 s (freestyle) and ~28 s (butterfly), and ~36 s (backstroke) and ~56 s (butterfly) for 200 m events. Inter-individual differences diminish in the later years, as evidenced by lower SD values relative to the remaining age groups and reported elsewhere [1,7,12]. Given swimming as an intermediate specialization sport (mean age at specialization ~13 years and starting age at ~7 [30]), the progression observed in female swimmers appears to follow the expected development pathway described in the literature [4]. A consistent decrease in performance improvements throughout consecutive ages from 9–10 to 20–21 years seems to occur across all freestyle distances in both sexes [11,15]. However, a trend toward (non)fluctuations was found during early junior age (i.e., 14 years) or junior age (i.e., 16 years) and close to the performance peak [1,11]. A greater improvement was found between the ages of 10 and 15 (~2–13% per year) and a decrease after 15–16 years (~1% per year). This pattern shows a relative age effect in childhood and aligns with other countries (e.g., Germany [31]). This pattern appears to be unaffected by sex [11] and is common to all events (i.e., distances and techniques [20]). Despite this, it should be noted that World Aquatics Points Scoring for female swimmers ranged from 627.10 ± 38.78 (50 m breaststroke) to 722.36 ± 35.86 (200 m freestyle). Based on Ruiz-Navarro et al.’s [23] classification model, female swimmers were within level 2, indicating a competitive yet not top-elite profile. However, a distinct pattern for level 2 swimmers was found [11]. Here, they showed the smallest improvements during early junior age, but continued to progress just before peak performance age. In addition, female swimmers with the highest expertise demonstrate better performance from ages 12–13 in 100 m freestyle [14]. Lower-level peers also exhibited substantial skill development from age 14 [14], aligning with the broader maturational convergence observed by Ruiz-Navarro and Born [11]. It could be argued that the mixed findings lie in the type of analysis (i.e., freestyle only vs. different swimming techniques).

Although annual progression and improvements throughout consecutive ages provide valuable insights for coaches and swimmers, the stability goes further into screening performance changes and accurate predictions. Notwithstanding, the mean stability trends found in the current cohort (i.e., 15 years for all 50 m events, 100 m freestyle, 200 m backstroke, breaststroke and butterfly; 16 years for 100 m backstroke, breaststroke and butterfly, and 200 m freestyle) are still controversial. Previous findings demonstrated an earlier plateau in female swimmers [7], since Portuguese male swimmers’ performance tended to stabilize also at 15 years [12]. These mixed findings may be related to contextual factors, such as expertise discrepancies or cultural differences influencing developmental patterns [6]. Despite the pattern of progression, the 200 m backstroke and butterfly showed differences for 15–16 years, which suggests later-maturing peers or, possibly, later menarche due to intensive training [4] for this distance. Collectively, this initial stage of improvement in girls’ swimming careers is a multifactorial phenomenon, primarily related to experienced anthropometric growth during the maturity phases [18,32]. Consequently, it leads to increased technical skills [33] being translated into more efficient patterns (i.e., greater in-water forces [34]). This also allows a clearer interpretation of variations across mean and normative stabilities, since rank-order shifts are expected due to inter-individual differences in maturational development.

The decline in SD of absolute values suggests that inter-individual variability in performance times decreases with age, reflecting narrower dispersion and greater homogeneity within the group’s performance trajectory. This reduction aligns with maturational dynamics, where early-maturing athletes initially gain an advantage and exhibit more stable trajectories. Conversely, late maturers progressively converge or even surpass them during later adolescence [1,15,33]. Conversely, results of normative stability contrast markedly with these mean findings. Intra-individual patterns of change appear to stabilize only in later adolescence (i.e., around ages 15–16), when predictive accuracy significantly increases [23]. The observed trend of increasing diagonal correlations for yearly performances aligns with previous evidence demonstrating similar patterns of stability over time. This distance- and technique-specific variability underscores that developmental pathways are not uniform, differing by specialization and maturational timing [14,17,22]. Overall, while mean stability reflects maturationally driven group progress, normative analyses reveal significant heterogeneity in yearly developmental trajectories, thus limiting the predictive capacity of early performances (i.e., not uniform, but rather highly dependent on both the swimmer’s age and the specific event). While existing literature on Olympic swimmers has attempted to compare competitive events [29], these comparisons often rely on indirect, non-statistical assessments. Furthermore, previous studies have focused on the specific niche of Olympic semi-finalists. Consequently, a direct statistical comparison across competitive events in the broader population of female swimmers has been lacking. This data analysis demonstrates that, once age, distance, and technique are accounted for, the remaining variance attributable to the swimmers themselves remains highly informative by providing a controlled comparison of how stability patterns differ across specific competitive events.

Moreover, the mixed-effects models demonstrated that age, distance and technique influenced performance stability, with distance showing the most substantial effect. The high R^2^c values reflect the strong explanatory power of the model, primarily driven by age-related development and event characteristics. In practical terms, this means that early performance is a poor predictor of long-term standing because the variance due to a certain swimmer is small compared to the variance driven by the rapid, non-linear improvements associated with maturation. While backstroke (ICC = 0.13) was the technique that showed the greatest rank-order stability across ages, the 50 m events (ICC = 0.15) were the ones that showed such stability. These findings suggest that different approaches are required across events, with some demanding a more flexible and long-term perspective in talent identification. Although ICC results should be carefully interpreted due to their weak coefficient levels, they suggest that short distances and specific techniques may yield higher consistency of individual differences and their stability in developmental trajectories. Likewise, mean stability analyses proved that all 50 m events present stability by age 15, whereas some 100 m (backstroke, breaststroke and butterfly) and 200 m (freestyle and butterfly) distances were achieved later at 16 years (except for 200 m backstroke, which stabilized at 14 years). Conversely, an indirect analysis across different events showed that female swimmers reach their threshold of peak performance in longer distance events at a younger age [15]. These divergences of findings may be translated through the inclusion of medley events or related to differences in methodological approach (no direct comparison across events using LMM was performed).

These findings are partly in line with previous evidence suggesting that the biomechanical and physiological demands of short-, middle-, and long-distance events may not strongly affect performance fluctuations within each competition season [1,7,11]. Extending this perspective from distance to technique specialization, energetic differences between swimming techniques may also account for variability. Freestyle and backstroke appear to be the most economical techniques in international swimmers, followed by butterfly and breaststroke [35]. In addition, backstroke is one of the introductory learn-to-swim techniques, allowing more time to develop technical skills, which in turn may contribute to their earlier inter-group stability [7] and reinforcing the need to promote within-sport specialization perspectives [36]. These biomechanical, physiological and pedagogical distinctions may help explain the slightly greater stability observed in short-distance and backstroke performances found in the present cohort.

Overall, the data of this study relates to the Portuguese context and may be carefully addressed to the remaining countries. To counteract discouragement from performance plateaus, coaches must actively use the quantified annual progression data provided in this study in combination with percentile curves and mathematical models [37] to establish appropriate goals and ensure realistic expectations as swimmers approach peak performance age. Moreover, frameworks addressing the different developmental phases and normative benchmarks are welcome to be integrated into this analysis.

In the end, some limitations can be addressed due to methodological approach: (i) the retrospective design without merging with developmental stages may have left some trajectory nuances out, such as vulnerability to biological spurts of young swimmers or later performances; (ii) the annual performance progression also depends on coaching systems and training characteristics, which differ between swimming squads; and (iii) only Top-50 Portuguese female swimmers were considered, which requires caution when trying to generalize data to broader samples due to lack of external validity. Although the raised points may lead to a small selection bias, the scientific approach and methods used here are perfectly aligned with those from previous studies that analyzed performance progression trajectories. Future research should focus on different analyses during the swimmer’s career, such as the tracking of maintenance in rankings during different chronological ages, which would provide complementary perspectives. Moreover, the use of larger samples expanding to other countries could be beneficial to understand how cultural and socioeconomic differences may be reflected in stability-related behaviors.

## 5. Conclusions

Performance improvements were most pronounced in the younger age groups, with progressively smaller gains approaching adulthood. Taken together, these findings allow us to make conclusions for swimmers’ long-term development: (1) interpreting early success or attempting to identify talented female Portuguese swimmers before the age of 15 years must be avoided; (2) despite low stability patterns and reduced variations across events, the backstroke technique demonstrated slightly greater consistency of individual differences on developmental trajectories, whereas shorter races (i.e., 50 m) tended to be more stable; and (3) distance appears to exert a stronger influence on stability than technique.

## Figures and Tables

**Table 1:** Mean ± SD percentage (%) changes in swimming performance between consecutive ages (10–18 years) and overall career.

Event		Changes Between Consecutive Years (%)								Overall (%)
		10–11	11–12	12–13	13–14	14–15	15–16	16–17	17–18	10–18
Freestyle	50 m	8.66 ± 4.90	5.43 ± 3.11	4.52 ± 3.02	2.35 ± 2.34	3.61 ± 3.07	0.86 ± 2.09	−0.53 ± 2.00	0.69 ± 1.72	23.96 ± 6.01
	100 m	10.50 ± 5.17	6.05 ± 2.60	3.54 ± 2.39	2.55 ± 1.81	1.58 ± 2.38	1.17 ± 2.23	0.75 ± 2.06	−0.39 ± 2.66	24.63 ± 6.68
	200 m	12.16 ± 5.57	7.36 ± 3.72	3.90 ± 2.88	3.01 ± 2.82	2.10 ± 1.96	1.49 ± 2.34	0.75 ± 1.98	0.10 ± 1.79	28.14 ± 7.10
Backstroke	50 m	9.13 ± 4.16	7.32 ± 3.69	3.79 ± 2.59	3.77 ± 3.12	2.39 ± 3.20	1.58 ± 2.18	0.39 ± 3.38	0.36 ± 2.06	26.96 ± 3.99
	100 m	11.02 ± 3.57	6.58 ± 2.59	4.54 ± 2.37	2.61 ± 1.96	1.54 ± 2.10	1.52 ± 3.15	0.47 ± 2.55	0.49 ± 2.56	26.81 ± 4.28
	200 m	6.73 ± 2.27	5.50 ± 2.08	4.12 ± 3.13	2.61 ± 2.27	1.36 ± 2.11	1.91 ± 2.09	0.37 ± 3.17	−0.25 ± 2.77	22.36 ± 4.47
Breaststroke	50 m	9.30 ± 4.34	8.20 ± 3.81	4.19 ± 3.00	3.26 ± 3.08	3.92 ± 2.30	1.06 ± 2.32	0.92 ± 2.44	−0.60 ± 3.46	28.60 ± 5.45
	100 m	8.92 ± 4.42	6.49 ± 2.59	4.42 ± 3.12	3.35 ± 2.46	2.63 ± 2.55	1.29 ± 2.01	1.60 ± 2.92	0.11 ± 3.48	26.68 ± 5.49
	200 m	8.55 ± 3.80	6.84 ± 3.23	4.10 ± 3.12	3.02 ± 3.59	2.54 ± 2.46	1.46 ± 2.65	0.57 ± 1.92	−0.38 ± 3.07	25.63 ± 6.24
Butterfly	50 m	9.65 ± 4.44	9.03 ± 4.01	5.02 ± 5.26	4.27 ± 2.95	3.18 ± 1.96	1.57 ± 1.83	0.48 ± 2.57	−0.37 ± 2.40	30.04 ± 7.19
	100 m	13.85 ± 5.05	8.83 ± 3.81	4.36 ± 3.58	3.04 ± 3.31	2.11 ± 2.92	1.50 ± 2.49	0.45 ± 1.77	0.04 ± 2.21	30.89 ± 7.47
	200 m	9.31 ± 3.65	9.07 ± 2.84	4.95 ± 2.96	4.63 ± 3.46	2.14 ± 1.37	2.38 ± 2.76	0.32 ± 3.07	−0.39 ± 2.72	29.90 ± 6.55

Note: Red cells indicate the age intervals where performance stability was reached (i.e., *p* > 0.05).

**Table 2:** Mixed-effect model results for swimming performance separated by distance and technique, including linear mixed model results for age, technique, and distance effects.

Mixed-Effect Model				
**Distance**	**R^2^c**	**ICC**	**F, *p*-value _age_**	**F, *p*-value _technique_**
50 m	0.87	0.15	350.29, *p* < 0.001	652.15, *p* < 0.001
100 m	0.86	0.12	644.22, *p* < 0.001	982.47, *p* < 0.001
200 m	0.86	0.12	485.29, *p* < 0.001	794.53, *p* < 0.001
**Technique**	**R^2^c**	**ICC**	**F, *p*-value _age_**	**F, *p*-value _distance_**
Freestyle	0.97	0.00	135.47, *p* < 0.001	13,539.63, *p* < 0.001
Backstroke	0.99	0.13	244.39, *p* < 0.001	30,652.96, *p* < 0.001
Breaststroke	0.98	0.11	180.88, *p* < 0.001	18,507.15, *p* < 0.001
Butterfly	0.97	0.08	153.62, *p* < 0.001	11,345.75, *p* < 0.001

Notes: F—F-test; ICC—intraclass correlation coefficient; R^2^c—conditional r squared.

**Figure A1:**
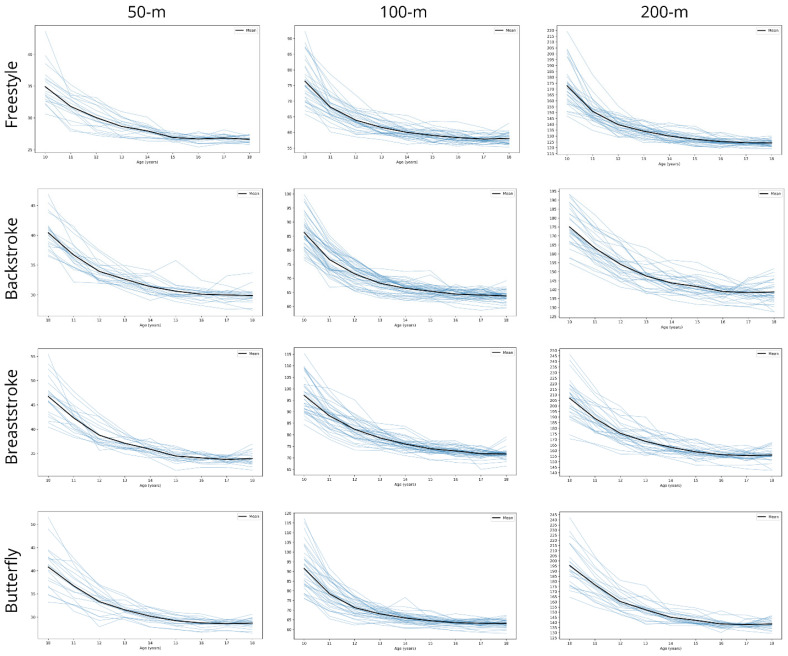
Spaghetti plots (light blue lines) of individual female swimmers’ performances across ages 10–18 years for each competitive event, with the mean trajectory shown in dark blue.

## Data Availability

The original data presented in the study are openly available in the SwimRankings database at http://www.swimrankings.net (accessed on 17 July 2025).

## Supplementary Materials

The following supporting information can be downloaded at https://www.mdpi.com/article/10.3390/sports14040164/s1, Table S1: Performance distributions of female swimmers across ages 10–18 years for each competitive event. Mean ± SD performance (s) and 95% CI are shown for each age, while repeated-measures ANOVA is given for each event.

## References

[1] Costa M.J., Marinho D.A., Bragada J.A., Silva A.J., Barbosa T.M. (2011). Stability of elite freestyle performance from childhood to adulthood. J. Sports Sci..

[2] Costa M.J., Quinta-Nova L., Ferreira S., Costa A.M., Santos C.C. (2024). Trend forecasting in swimming world records and in the age of world record holders. Appl. Sci..

[3] Brustio P.R., Cardinale M., Lupo C., Varalda M., De Pasquale P., Boccia G. (2021). Being a top swimmer during the early career is not a prerequisite for success: A study on sprinter strokes. J. Sci. Med. Sport.

[4] Malina R.M., Rogol A.D., Cumming S.P., Coelho e Silva M.J., Figueiredo A.J. (2015). Biological maturation of youth athletes: Assessment and implications. Br. J. Sports Med..

[5] Towlson C., Cobley S., Parkin G., Lovell R. (2018). When does the influence of maturation on anthropometric and physical fitness characteristics increase and subside?. Scand. J. Med. Sci. Sports.

[6] Baker J., Horton S., Robertson-Wilson J., Wall M. (2003). Nurturing sport expertise: Factors influencing the development of elite athlete. J. Sports Sci. Med..

[7] Born D.P., Lomax I., Rüeger E., Romann M. (2022). Normative data and percentile curves for long-term athlete development in swimming. J. Sci. Med. Sport.

[8] Fitrianto A.T., Rizky O.B., Rahmadi E., Ramadhan A. (2025). A systematic literature review of swimming performance prediction: Methods, datasets, techniques and research trends. Retos.

[9] Allen S.V., Hopkins W.G. (2015). Age of peak competitive performance of elite athletes: A systematic review. Sports Med..

[10] Barreiros A., Côté J., Fonseca A.M. (2014). From early to adult sport success: Analysing athletes’ progression in national squads. Eur. J. Sport Sci..

[11] Ruiz-Navarro J.J., Born D.P. (2025). Annual performance progression in swimming across competition levels and race distances. J. Funct. Morphol. Kinesiol..

[12] Costa M.J., Marinho D.A., Reis V.M., Silva A.J., Marques M.C., A Bragada J., Barbosa T.M. (2010). Tracking the performance of world-ranked swimmers. J. Sports Sci. Med..

[13] Morais J.E., Costa M.J., Forte P., Marques M.C., Silva A.J., Marinho D.A., Barbosa T.M. (2014). Longitudinal intra- and inter-individual variability in young swimmers’ performance and determinant competition factors. Mot. Rev. Educ. Fis..

[14] Post A.K., Koning R.H., Visscher C., Elferink-Gemser M.T. (2020). Multigenerational performance development of male and female top-elite swimmers–A global study of the 100 m freestyle event. Scand. J. Med. Sci. Sports.

[15] Dormehl S.J., Robertson S.J., Williams C.A. (2016). How confident can we be in modelling female swimming performance in adolescence?. Sports.

[16] Kuberski M.J., Musial A., Krużołek P., Błażkiewicz M., Wąsik J., Konarski J. (2025). Determinants of pre-adolescent girls’ sport performance in three-year swimming training. Front. Sports Act. Living.

[17] Born D.P., Stöggl T., Lorentzen J., Romann M., Björklund G. (2024). Predicting future stars: Probability and performance corridors for elite swimmers. J. Sci. Med. Sport.

[18] Malina R.M. (2001). Adherence to physical activity from childhood to adulthood: A perspective from tracking studies. Quest.

[19] Morais J.E., Barbosa T.M., Forte P., Bragada J.A., Castro F.A.d.S., Marinho D.A. (2023). Stability analysis and prediction of pacing in elite 1500 m freestyle male swimmers. Sports Biomech..

[20] Pyne D., Trewin C., Hopkins W. (2004). Progression and variability of competitive performance of Olympic swimmers. J. Sports Sci..

[21] Asendorpf J.B., Valsiner J. (1992). Stability and Change in Development: A Study of Methodological Reasoning.

[22] Born D.P., Lomax I., Horvath S., Meisser E., Seidenschwarz P., Burkhardt D., Romann M. (2020). Competition-based success factors during the talent pathway of elite male swimmers. Front. Sports Act. Living.

[23] Ruiz-Navarro J.J., López-Belmonte Ó., Gay A., Cuenca-Fernández F., Arellano R. (2023). A new model of performance classification to standardize the research results in swimming. Eur. J. Sport Sci..

[24] Swimrankings.net. http://www.swimrankings.net.

[25] World Aquatics Swimming Rules and Swimming Points. https://www.worldaquatics.com/rules/.

[26] Bragada J.A., Santos P.J., Maia J.A., Colaço P.J., Lopes V.P., Barbosa T.M. (2010). Longitudinal study in 3000 m male runners: Relationship between performance and selected physiological parameters. J. Sports Sci. Med..

[27] Kowalski C.J., Schneiderman C.D. (1992). Tracking: Concepts, methods and tools. Int. J. Anthropol..

[28] Ferguson C.J. (2009). An effect size primer: A guide for clinicians and researchers. Prof. Psychol. Res. Pract..

[29] Allen S.V., Vandenbogaerde T.J., Hopkins W.G. (2014). Career performance trajectories of Olympic swimmers: Benchmarks for talent development. Eur. J. Sport Sci..

[30] De Bosscher V., Descheemaeker K., Shibli S. (2023). Starting and specialisation ages of elite athletes across Olympic sports: An international cross-sectional study. Eur. J. Sport Sci..

[31] Staub I., Stallman R.K., Vogt T. (2020). The relative age effect in German 11- to 18-year-old male and female swimmers. Ger. J. Exerc. Sport Res..

[32] Gulbin J.P., Croser M.J., Morley E.J., Weissensteiner J.R. (2013). An integrated framework for the optimisation of sport and athlete development: A practitioner approach. J. Sports Sci..

[33] Saavedra J.M., Escalante Y. (2010). A multivariate analysis of performance in young swimmers. Pediatr. Exerc. Sci..

[34] Santos C.C., Marinho D.A., Costa M.J. (2024). Changes in young swimmers’ in-water force, performance, kinematics, and anthropometrics over a full competitive season. J. Hum. Kinet..

[35] Barbosa T.M., Fernandes R., Keskinen K.L., Colaço P., Cardoso C., Silva J., Vilas-Boas J.P. (2006). Evaluation of the energy expenditure in competitive swimming strokes. Int. J. Sports Med..

[36] Staub I., Zinner C., Bieder A., Vogt T. (2020). Within-sport specialisation and entry age as predictors of success among age group swimmers. Eur. J. Sport Sci..

[37] Morais J.E., Forte P., Silva A.J., Barbosa T.M., Marinho D.A. (2021). Data modeling for inter- and intra-individual stability of young swimmers’ performance: A longitudinal cluster analysis. Res. Q. Exerc. Sport.

