# Supplementary material for: Performance Progression and Stability of Female Swimmers Across Different Swimming Techniques from Childhood to Adulthood
Source: Sports (Basel). 2026 Apr 21;14(4):164. doi: 10.3390/sports14040164 (PMC13120615; doi:10.3390/sports14040164)
Supplement: Supplementary file 1 [file sports-14-00164-s001.zip › sports-4206859-supplementary.pdf]

Table S1. Performance distributions of female swimmers across ages 10–18 years for each competitive event. Mean ± SD performance (s) and 95% CI are shown for each age, while repeated-measures ANOVA is given for each event

Event		ANOVA	10	11	12	13	14	15	16	17	18
Freestyle	**50-m (n=19)**	F(1.81, 32.61)=103,21, p<0.001, η^2=0.85	34.92 ± 3.12 [33.42, 36.42]	31.80 ± 2.06 [30.81, 32.80]	30.05 ± 1.83 [29.17, 30.93]	28.65 ± 1.24 [28.05, 29.25]	27.96 ± 0.93 [27.51, 28.41]	26.93 ± 0.46 [26.71, 27.15]	26.69 ± 0.59 [26.41, 26.98]	26.83 ± 0.58 [26.55, 27.11]	26.64 ± 0.49 [26.40, 26.87]
	**100-m (n=33)**	F(1.69, 54.18)=172.48, p<0.001, η^2=0.84	76.37 ± 6.90 [73.92, 78.81]	68.08 ± 3.95 [66.68, 69.48]	63.89 ± 2.90 [62.86, 64.92]	61.59 ± 2.16 [60.82, 62.35]	60.00 ± 1.89 [59.33, 60.67]	59.03 ± 1.77 [58.41, 59.66]	58.33 ± 1.61 [57.75, 58.90]	57.87 ± 1.32 [57.40, 58.34]	58.08 ± 1.73 [57.47, 58.70]
	**200-m (n=33)**	F(1.49, 47.58)=187.24, p<0.001, η^2=0.85	172.81 ± 17.52 [166.59, 179.02]	151.00 ± 8.86 [147.85, 154.14]	139.69 ± 6.31 [137.45, 141.93]	134.13 ± 4.74 [132.45, 135.80]	130.04 ± 4.61 [128.41, 131.67]	127.27 ± 4.15 [125.80, 128.74]	125.31 ± 3.12 [124.21, 126.42]	124.33 ± 2.27 [123.52, 125.13]	124.19 ± 2.57 [123.28, 125.10]
Backstroke	**50-m (n=21)**	F(3.06, 61, 17)=167.29, p<0.001, η^2=0.89	40.45 ± 2.81 [39.16, 41.73]	36.70 ± 2.32 [35.64, 37.75]	33.96 ± 1.62 [33.22, 34.69]	32.64 ± 1.17 [32.11, 33.17]	31.40 ± 1.30 [30.81, 31.99]	30.63 ± 1.40 [30.00, 31.27]	30.13 ± 0.92 [29.71, 30.55]	30.00 ± 1.15 [29.48, 30.52]	29.89 ± 1.31 [29.30, 30.49]
	**100-m (n=42)**	F(2.37, 97.29)=384.60, p<0.001, η^2=0.90	86.29 ± 5.96 [84.43, 88.15]	76.68 ± 4.59 [75.25, 78.11]	71.56 ± 3.55 [70.45, 72.66]	68.26 ± 2.62 [67.44, 69.07]	66.45 ± 2.36 [65.72, 67.19]	65.41 ± 2.25 [64.71, 66.11]	64.37 ± 1.75 [63.82, 64.91]	64.05 ± 1.83 [63.48, 64.62]	63.72 ± 1.99 [63.10, 64.34]
	**200-m (n=30)**	F(2.42, 70.26)=187.51, p<0.001, η^2=0.87	175.05 ± 10.61 [171.09, 179.02]	163.13 ± 8.36 [160.01, 166.25]	154.10 ± 7.65 [151.25, 156.96]	147.61 ± 6.04 [145.36, 149.87]	143.69 ± 5.27 [141.73, 145.66]	141.71 ± 5.13 [139.79, 143.62]	138.94 ± 4.17 [137.39, 140.50]	138.36 ± 4.18 [136.80, 139.92]	138.72 ± 6.12 [136.43, 141.01]
Breaststroke	**50-m (n=20)**	F(2.18, 41.34)=140.05, p<0.001, η^2=0.88	46.74 ± 4.13 [44.81, 48.68]	42.27 ± 2.60 [41.05, 43.49]	38.76 ± 2.23 [37.72, 39.80]	37.08 ± 1.30 [36.47, 37.69]	35.85 ± 1.09 [35.34, 36.36]	34.44 ± 1.21 [33.87, 35.00]	34.06 ± 0.89 [33.64, 34.47]	33.73 ± 0.73 [33.39, 34.07]	33.93 ± 1.37 [33.29, 34.57]
	**100-m (n=37)**	F(2.12, 76.45)=251.89, p<0.001, η^2=0.88	97.09 ± 7.81 [94.48, 99.69]	88.21 ± 5.41 [86.41, 90.02]	82.43 ± 4.49 [80.93, 83.93]	78.69 ± 2.98 [77.69, 79.68]	76.02 ± 2.67 [75.13, 76.91]	73.98 ± 2.02 [73.30, 74.65]	73.00 ± 1.98 [72.34, 73.67]	71.80 ± 2.03 [71.13, 72.48]	71.69 ± 2.23 [70.95, 72.43]
	**200-m (n=29)**	F(2.18, 61,05)=166.89, p<0.001, η^2=0.86	206.86 ± 17.62 [200.16, 213.56]	188.77 ± 12.77 [183.91, 193.63]	175.62 ± 9.90 [171.86, 179.39]	168.29 ± 8.59 [165.02, 171.56]	162.98 ± 5.62 [160.85, 165.12]	158.80 ± 5.66 [156.65, 160.95]	156.38 ± 4.11 [154.82, 157.94]	155.47 ± 4.34 [153.82, 157.12]	156.03 ± 5.87 [153.80, 158.26]
Butterfly	**50-m (n=19)**	F(1.60, 28.75)=115,88, p<0.001, η^2=0.87	40.82 ± 4.73 [38.53, 43.10]	36.75 ± 3.44 [35.10, 38.41]	33.36 ± 2.61 [32.10, 34.62]	31.58 ± 1.52 [30.85, 32.31]	30.21 ± 1.27 [29.60, 30.82]	29.24 ± 1.05 [28.73, 29.74]	28.78 ± 1.06 [28.26, 29.29]	28.62 ± 0.69 [28.28, 28.95]	28.72 ± 1.04 [28.22, 29.23]
	**100-m (n=36)**	F(1.45, 50.76)=204.61, p<0.001, η^2=0.85	91.52 ± 11.07 [87.78, 95.26]	78.45 ± 6.75 [76.17, 80.74]	71.33 ± 4.30 [69.88, 72.79]	68.10 ± 2.69 [67.19, 69.01]	66.00 ± 2.92 [65.01, 66.99]	64.56 ± 2.14 [63.83, 65.28]	63.56 ± 1.68 [62.99, 64.12]	63.26 ± 1.85 [62.64, 63.89]	63.23 ± 1.95 [62.57, 63.89]
	**200-m (n=24)**	F(1.57, 36.20)=163.00, p<0.001, η^2=0.88	195.41 ± 20.04 [186.95, 203.87]	176.67 ± 13.00 [171.18, 182.16]	160.38 ± 8.88 [156.63, 164.13]	152.37 ± 8.68 [148.71, 156.04]	145.09 ± 5.06 [142.95, 147.23]	141.96 ± 4.46 [140.07, 143.84]	138.52 ± 4.34 [136.68, 140.35]	137.98 ± 3.19 [136.63, 139.33]	138.51 ± 4.61 [136.56, 140.45]
